# Deep learning velocity signals allow quantifying turbulence intensity

**DOI:** 10.1126/sciadv.aba7281

**Published:** 2021-03-17

**Authors:** Alessandro Corbetta, Vlado Menkovski, Roberto Benzi, Federico Toschi

**Affiliations:** 1 Department of Applied Physics, Eindhoven University of Technology, Eindhoven, Netherlands.; 2 Department of Mathematics and Computer Science, Eindhoven University of Technology, Eindhoven, Netherlands.; 3 Department of Physics, University of Rome Tor Vergata, Rome, Italy.; 4 CNR-IAC, Rome, Italy.

## Abstract

Turbulence, the ubiquitous and chaotic state of fluid motions, is characterized by strong and statistically nontrivial fluctuations of the velocity field, and it can be quantitatively described only in terms of statistical averages. Strong nonstationarities impede statistical convergence, precluding quantifying turbulence, for example, in terms of turbulence intensity or Reynolds number. Here, we show that by using deep neural networks, we can accurately estimate the Reynolds number within 15% accuracy, from a statistical sample as small as two large-scale eddy turnover times. In contrast, physics-based statistical estimators are limited by the convergence rate of the central limit theorem and provide, for the same statistical sample, at least a hundredfold larger error. Our findings open up previously unexplored perspectives and the possibility to quantitatively define and, therefore, study highly nonstationary turbulent flows as ordinarily found in nature and in industrial processes.

## Teaser

Deep learning velocity signals allow quantifying turbulence intensity from very short signals and with extremely high accuracy.

## INTRODUCTION

Turbulence is characterized by complex statistics of velocity fluctuations correlated over a wide range of temporal and spatial scales. These range from the integral scale, *L*, characteristic of the energy injection (with correlation time *T_L_*), to the dissipative scale, η ≪ *L*, characteristic of the energy dissipation due to viscosity (with correlation time τ_η_ ≪ *T_L_*). The intensity of turbulence directly correlates with the width of this range of scales, *L*/η or *T_L_*/τ_η_, commonly dubbed inertial range.

In statistically stationary, homogeneous and isotropic turbulence (HIT), the width of the inertial range, is well known to correlate with the Reynolds number, Re, defined as Re = *v*_rms_*L*/ν, where *v*_rms_ is the characteristic velocity fluctuation at the integral scale, and ν is the kinematic viscosity. In the presence of a mean velocity, turbulence intensity is defined as the root mean square velocity fluctuation, *v*_rms_, divided by the average velocity, $\bar{v}$. Therefore, once the average wind speed, $\bar{v}$, is known, the value of the Reynolds number can be used to quantify turbulence intensity and vice versa. While this value remains well defined in laboratory experiments, performed under stationary conditions, and for fixed flow configurations (*L* = const), its quantification is impossible when we consider turbulence in open environments (as in many outstanding geophysical situations) or in nonstationary situations (such as turbulent/nonturbulent interfaces). This observation is linked to the question: Can we estimate turbulence intensity from fluctuating velocity signals of arbitrary (short) length? For statistically stationary conditions, this is indeed possible provided that enough statistical samples are available and by using appropriate physics-based statistical averages of the fluctuating velocity field. For nonstationary turbulent flows (i.e., changing on time scales comparable with the large-scale correlation times), the question itself appears meaningless. In these conditions, the intertwined complexity of a slow large-scale dynamics and of fast, but highly intermittent, small-scale fluctuations, makes it impossible to reliably estimate the width of the inertial range.

Here, we demonstrate, using a proof of concept, that our fundamental question can be answered by a suitable use of machine learning. We propose a machine learning deep neural network (DNN) model capable of estimating turbulence intensity within 15% accuracy from short velocity signals (duration *T*: approximately two large-scale eddy turnover times, i.e., *T* ≈ 2 *T_L_*, where *T_L_* ≈ *L*/*v*_rms_). We remark that analyzing the same data via standard statistical observables of turbulence leads to quantitatively meaningless results (predictions between 10^−2^ and 10^2^ times the true value).

### DNN-based turbulence intensity measurements

We train the DNN model using (short) signals proxy for the Lagrangian velocity in HIT. The proxy Lagrangian velocity signals, *v*(*t*), that we use are obtained as the superposition of different strongly chaotic time signals, *u_n_*(*t*), derived from a shell model (SM) of turbulence (*1*, *2*) (see Methods). The velocity signals, *v*(*t*), obtained from such an SM are known to closely match the statistical properties of the velocity experienced by a passive Lagrangian particle in HIT (*3*). As Lagrangian velocities are one of the most intermittent features of turbulence, we are choosing the most difficult case for our proof of concept.

The SM describes the nonlinear energy transfer among different spatial scales, *l_n_* = 1/*k_n_*, where *k_n_* = *L*^−1^λ*^n^* (with *L*^−1^ = 0.05 being the wave number associated with the integral scale, and λ = 2 defining the ratio between successive shells). The nonlinear energy transfer is characterized by sudden bursts of activity (typically referred to as “instantons”) (*1*, *4*), where anomalous fluctuations are spread from large to small scales. The complex space-time patterns and localized correlations in *v*(*t*), given by these intermittent bursts, make a one-dimensional convolutional neural network (CNN) a well-suited choice for our neural network model (see Methods and the Supplementary Materials for details).

**Fig. 1:**
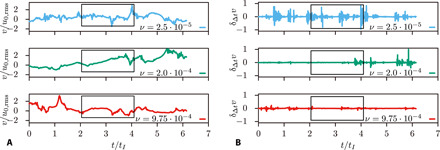
Velocity signals, *v*(*t*), generated by the SM used to train the DNN for three different values of the viscosity *ν*. (**A**) Velocity *v*(t), normalized with the root mean square of the integral-scale velocity, *v*_0,rms_ ≈ ν_rms_. The time is reported in units of eddy turnover times of the integral scale, *T_L_* (*T_L_* ≈ 1000Δ*t*, where Δ*t* is the time sampling of the DNN input signals). Each training signal spans 2048 samples, i.e., about two eddy turnover times (the rectangular frames identify individual training signals). (**B**) Velocity increments, δ_Δ*t*_ *v*(*t*) = *v*(*t* + Δ*t*) − *v*(*t*), computed with time interval Δ*t*. Lower viscosity values yield higher turbulence intensity, thus more intermittent high-frequency components and more intense small-scale velocity differences.

We train the DNN using a collection of datasets corresponding to different viscosity values for our SM. Each dataset includes a large number of (Lagrangian, turbulent) velocity signals (few thousands) sampled over 2048 time instants (see examples in Fig. 1A). We decided to use an external forcing to maintain the root mean square energy fluctuations of the signals, and thus *v*_rms_, statistically stationary. As a result, the viscosity fully determines the turbulence intensity and therefore the Reynolds number. Decreasing the viscosity increases the high-frequency content of the velocity signals (as ~Re^1/2^, cf. time increments in Fig. 1B) by reducing the dissipative time and length scales. The resulting wider inertial range reflects the higher turbulence intensity. We train the network in a supervised way to infer the viscosity from the velocity signals; the collection of datasets covered uniformly the viscosity interval 10^−5^ ≤ ν ≤ 10^−3^ in 39 equi-spaced levels.

## RESULTS

**Fig. 2:**
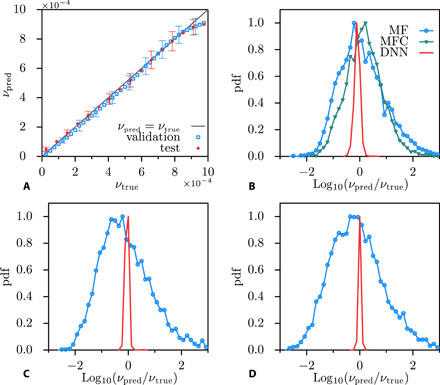
Neural network viscosity prediction performance and comparison with multifractal-based estimates. (**A**) Average predictions of viscosity, *ν*, by the DNN (*ν*_pred_, *y* axis) versus ground truth (*ν*_true_, *x* axis), for the validation and test sets considered. The diagonal line identifies error-free predictions, i.e., *ν*_pred_ = *ν*_true_. We include a few indicative error bars of size ±σ from the average, to indicate the typical spread of the prediction. (**B** to **D**) Comparison of the viscosity estimates over three viscosity levels in the validation set, respectively, *ν* = 0.000075, 0.0002, and 0.0007). We report the pdf of log_10_(*ν*_pred_/*ν*_true_) for the DNN (solid lines), and for the multifractal (MF) model (dotted lines), Eq. 5. Evaluating *v*_rms_ in Eq. 2 through an ensemble averaged (MF), or individually for each signal as $v_{\text{rms}}^{2}=\frac{1}{2}S_{2}(\infty )\approx \frac{1}{2}S_{2}(T)$ (MFC), yields similar results, which we report in (B). We notice how the predictions based on Eq. 5, once normalized to the true value, range within about four orders of magnitude, whereas they remain confined within 15% accuracy in the case of the DNN.

We assess the predictive performance of the DNN by considering unseen signals generated by the SM. Results are reported in Fig. 2 where two different test sets are used: (i) “validation set”: including statistically independent realizations of the velocity signals than in the training phase yet having the same viscosity values; (ii) “test set”: signals having different viscosity values than considered during training yet within the same viscosity range. The results demonstrate that the network is capable of very accurate predictions for the viscosity over the full range considered, also for viscosity values different from those used in training. By aggregating the viscosity estimates over a large number of statistically independent SM signals with fixed viscosity, we can define an average estimate as well as a root mean square error, (see Fig. 2A).

To further reflect on this remarkable result, we turn to a physical argument for estimating the viscosity. Defining the second-order Lagrangian structure functions *S*_2_(τ) = 〈δ_τ_*v*(*t*)^2^〉, where δ_τ_*v*(*t*) = *v*(*t* + τ) − *v*(*t*) and 〈…〉 is a time average, we consider the short-time limit of the averaged squared velocity gradients

$$\underset{\tau \to 0}{\text{lim}}\frac{S_{2}(\tau )}{\tau^{2}}=\langle {\left(\partial_{t}v\right)}^{2}\rangle$$ (1)

and we compare it with $v_{\text{rms}}^{2}$. The ratio of these two quantities scales as Re^1/2^ based on Kolmogorov 1941 dimensional theory and as Re^α^ where α ≈ 0.57 if one considers also intermittency corrections (see the Supplementary Materials for a derivation of the relation). Consistent with Eq. 1, it holds: $\underset{\tau \to 0}{\text{lim}}\frac{S_{2}(\tau )}{v_{\text{rms}}^{2}\tau^{2}}=ARe^{\alpha }$. Let us consider *N* Lagrangian velocity signals, *i* = 1,2, …, *N*, produced by the SM, having length 2*T_L_* (with *T_L_* the large-scale eddy turnover time) and with fixed viscosity $\hat{\nu }$ or, equivalently, Reynolds number $\hat{R}e$. For each signal, *i*, we compute the quantity

$$G_{i}\equiv \underset{\tau \to 0}{\text{lim}}\frac{1}{v_{\text{rms}}^{2}}\frac{\langle {\left(\delta v(\tau )\right)}^{2}\rangle }{\tau^{2}}$$ (2)

where the average 〈…〉 is computed over the signal *i*, thus for a time 2*T_L_*, and *v*_rms_ is evaluated over all the *N* samples (hence, it is an overall constant). Taking an average over the *N* samples, we know that

$$\bar{G}\equiv \frac{1}{N}\Sigma_{i}G_{i}=A\hat{R}e^{\alpha }$$ (3)

where *A* is a Re-independent and α ≈ 0.57. The quantity *A* can be thus computed via Eq. 3 given *N* ≫ 1. Now, for a signal *j* of unknown viscosity, we can compute the predicted Reynolds number Re_pred_(*j*) using Eq. 2, and for each *j* holds

$$Re_{\text{pred}}(j)=\hat{R}e{\left(\frac{G_{j}}{\bar{G}}\right)}^{\frac{1}{\alpha }}$$ (4)

Last, by means of Eq. 4, we predict the viscosity value, ν_pred_(*j*), of the signal *j* as

$$\nu_{\text{pred}}(j)={\left(\frac{\bar{G}}{G_{j}}\right)}^{\frac{1}{\alpha }}\hat{\nu }$$ (5)

Figure 2 (B to D) compares the probability density functions (PDFs) of the logarithmic ratio between the estimated and true viscosity (ν_pred_/ν_true_), for estimates by the DNN and based on Eq. 5. Predictions in case of Eq. 5 spread over a range ν_pred_/ν_true_ ∈ [10^−2^,10^2^], whereas this range is just of order 15% in case of the DNN. Besides, in Fig. 2B (“MFC” pdf), we observe that evaluating *v*_rms_ on a signal-by-signal basis reduces, yet minimally, the variance of viscosity predictions based on Eq. 5.

The high variance and heavy tails of the pdf of viscosity estimates produced by Eq. 5 follow from the very limited statistical sampling (2048 points), which is severely affected by large-scale oscillations and small-scale intermittent fluctuations. Because of these, statistical convergence and, therefore, a stable value for the right hand side (RHS) of Eq. 2 are attained only after very long observation times.

**Fig. 3:**
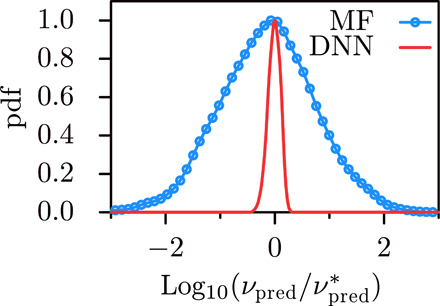
Comparison of the viscosity estimates by the DNN [trained through SM data, solid line] and by the MF model (dotted line) for Lagrangian velocity signals obtained by a DNS simulation. We normalize the estimates by the most frequent prediction, $\nu_{\text{pred}}^{*}$, i.e., we report the pdf of ${\text{log}}_{10}(\nu_{\text{pred}}/\nu_{\text{pred}}^{*})$. This enables a comparison of the prediction root mean square errors. As in the considered validation and test cases, the DNN estimates fall within a significantly smaller range than in case of predictions by the MF model. Note that a log-normal distribution is expected for the viscosity estimates (*10*), evident in the MF case.

Our DNN model can be tested on real Lagrangian velocity signals, *v*(*t*), obtained by the numerical integration of the Lagrangian dynamics of a tracer particle in a direct numerical simulation (DNS) of HIT, see Fig. 3. The underlying Eulerian velocity field is obtained from DNS (*5*) of the Navier-Stokes equation at Re_λ_ = 400 (see Methods). The DNN, although trained on SM data, is able to estimate with extremely high accuracy the viscosity ν even in case of real Lagrangian data [note that Lagrangian velocity signals from DNS have been exhaustively validated against experimental data in the past (*6*)]. This points to the fact that the DNN relies on space-time features that are equally present in the SM as well as in the real Lagrangian signals.

## DISCUSSION

What is the best result that can be achieved according to the current understanding of the physics of turbulence? Both by direct estimation of the Reynolds number and by viscous scale fitting, i.e., using Eq. 5, the statistical accuracy is limited by the fluctuations of the large-scale velocity. Therefore, the statistical error is limited by the number of large-scale eddy turnover times. As shown in Fig. 2 (B to D), a traditional statistical physics approach produces estimates for the viscosity spread over four orders of magnitude, while the DNN is capable of delivering accurate predictions, scattering within a 15% range.

This points at two major results: First, the DNN, at least within the range of the training signals, must be able to identify space-time structures that strongly correlate with turbulence intensity and which are rather insensitive to the strong fluctuations of the instantaneous value of the large-scale velocity (cf. the Supplementary Materials for a discussion). This finding unlocks the possibility of defining, practically instantaneously, turbulence intensities, Reynolds numbers, or connected statistical quantities for complex flows and fluids. Estimating locally, in space and in time, the turbulence intensity at laminar-turbulent interfaces or from atmospheric anemometric readings can now be possible. The quantitative definition of an effective viscosity within boundary layers or complex fluids, such as emulsions or viscoelastic flows, may similarly be pursued. Last, being able to extract the space-time correlations identified by the DNN may give us novel and fundamental insights in turbulence physics and the complex skeleton of the fluctuating turbulent energy cascades.

## METHODS

### Generating the database of turbulent velocity signals

We use the SABRA (*2*) SM of turbulence to generate Lagrangian velocity signals $v(t)=\sum_{n\geq 0}Ru_{n}(t)$ corresponding to different turbulence levels (Reynolds numbers). SMs evolve in time, *t* > 0, the complex amplitude of velocity fluctuations, *u_n_*(*t*), at logarithmically spaced wavelengths, *k_n_* = *k*_0_λ*^n^* (*n* = 0,1, …).

The amplitudes *u_n_*(*t*) evolve according to the following equation

$$\begin{array}{c} \frac{du_{n}(t)}{\mathrm{dt}}=i(ak_{n+1}u_{n+2}u_{n+1}^{*}+bk_{n}u_{n+1}u_{n-1}^{*}-ck_{n-1}u_{n-1}u_{n-2})\\ -\nu k_{n}^{2}u_{n}+f_{n}(t) \end{array}$$ (6)

where ν > 0 represents the viscosity; *f_n_*(*t*) is the forcing; and the real coefficients *a*, *b*, and *c* regulate the energy exchange between neighboring shells. We consider the following constraints: *a* + *b* + *c* = 0, which guarantees conservation of energy *E* = Σ*_n_*∣*u_n_*∣^2^, for an unforced and inviscid system (*f_n_* = 0, ν = 0, respectively); *b* = −1/2, which gives to the second (inviscid/unforced) quadratic invariant of the system, *H* = Σ_*n* ≥ 0_( − )*^n^k_n_*∣*u_n_*∣^2^, the dimensions of an helicity; to fix the third parameter, we opt for the common choice *c* = 1/2. We truncate Eq. 6 to a finite number of shells 0 ≤ *n* < *N* = 28, which ensures a full resolution of the dissipative scales in combination with our forcing and viscosity range. We simulate the system in Eq. 6 via a fourth-order Runge-Kutta scheme with viscosity explicitly integrated (*7*) (the integration step, *dt*, is fixed for all simulations, to be about three orders of magnitude smaller than the dissipative time scale for the lowest viscosity case).

We inject energy through a large-scale forcing acting on the first two shells (*2*). The forcing dynamics is given by an Ornstein-Uhlenbeck process with a time scale matching the eddy turnover of the forced shells (τ*_n_* = (*k_n_u_n_*), *n* = 0,1). In addition, we set the ratio $|\sigma (f_{0})/\sigma (f_{1})|=\sqrt{2}$ between the SD (σ(*f_n_*)) of the two forcing signals. This ensures a helicity-free energy flux in the system (*2*). See the Supplementary Materials for further information on the signals and values of the constants.

We generate the signals in a vectorized fashion on an NVIDIA V100 card. We integrate simultaneously 15,000 instances of the system in Eq. 6 in a vectorized manner (i.e., system description by 15,000 × 28 complex variables) and dump the state 55,000 times after skipping the first 5000 samples.

### Lagrangian velocity signals from DNSs

The true Lagrangian velocity signals are obtained from the numerical integration of Lagrangian tracer dynamics evolved on top of a DNS of HIT turbulence. The Eulerian flow field is evolved via a fully de-aliased algorithm with second-order Adams-Bashforth time stepping with viscosity explicitly integrated. The Lagrangian dynamics is obtained via a trilinear interpolation of the Eulerian velocity field coupled with second-order Adams-Bashforth integration in time. The Eulerian simulation has a resolution of 2048^3^ grid points, a viscosity of 3.5 · 10^−4^, and a time step *dt* = 1.2 · 10^−4^, this corresponded to a Re_λ_ ∼ 400, dissipative scale η = 3 · 10^−3^, and τ_η_ = 2 · 10^−2^. The Lagrangian trajectories used are available at the 4TU.Centre for Research Data (*8*).

### Deep neural network

We use a one-dimensional CNN architecturally inspired by the VGG model (*9*). Developing a neural network model poses the major challenge of selecting a large number of hyperparameters. This particular architecture deals with this issue by fixing the size of the filters and uses stacks of convolutional layers to achieve complex detectors. For our model, we opted for convolutional filters of size 3, which is comparable or smaller than the dissipative time scale of the turbulent signals. The network includes four blocks, each formed by three convolutional layers (including 128 filters each), a max pooling layer (window: 2), and a dropout layer, that capture all the spatial features of the signal (cf. DNN architecture in the Supplementary Materials). These layers are followed by a fully connected layer with 128 neurons and Rectified Linear Unit (Re-Lu) activation that collects all the spatial features into a dense representation. The final layer provides a linear map from the dense representation to the estimated viscosity. A complete sketch of the network is in the Supplementary Materials.

### DNN training

We train the neural network in a supervised fashion and with *L*^2^ training loss to output a continuous value in the interval [−1,1], which is linearly mapped to [min ν, max ν]. The training set is composed of 192,000 turbulent velocity signals (time-sampled over 2048 points) uniformly distributed among 39 viscosity levels (training-validation ratio, 75 to 25%). See table S1 for further information.

## Supplementary Material

http://advances.sciencemag.org/cgi/content/full/7/12/eaba7281/DC1

Adobe PDF - aba7281_SM.pdf

Deep learning velocity signals allow quantifying turbulence intensity

## SUPPLEMENTARY MATERIALS

Supplementary material for this article is available at http://advances.sciencemag.org/cgi/content/full/7/12/eaba7281/DC1
