# Supplementary material for: Deep learning velocity signals allow quantifying turbulence intensity
Source: Sci Adv. 2021 Mar 17;7(12):eaba7281. doi: 10.1126/sciadv.aba7281 (PMC7968843; doi:10.1126/sciadv.aba7281)
Supplement: http://advances.sciencemag.org/cgi/content/full/7/12/eaba7281/DC1 [file supp_7_12_eaba7281__7.12.eaba7281.DC1.html]

Science Advances | Science AdvancesAAASSearchScience AdvancesMenu

## Supplementary Materials

# Deep learning velocity signals allow quantifying turbulence intensity

Alessandro Corbetta, Vlado Menkovski, Roberto Benzi, Federico Toschi

Download Supplement

**This PDF file includes:**

- Sections S1 to S4
- Figs. S1 to S6
- Table S1

**Files in this Data Supplement:**

- Adobe PDF - aba7281\_SM.pdf
