# Supplementary material for: Deep learning velocity signals allow quantifying turbulence intensity
Source: Sci Adv. 2021 Mar 17;7(12):eaba7281. doi: 10.1126/sciadv.aba7281 (PMC7968843; doi:10.1126/sciadv.aba7281)
Supplement: http://advances.sciencemag.org/cgi/content/full/7/12/eaba7281/DC1 [file supp_7_12_eaba7281__aba7281_SM.pdf]

Science Advances
AAAS

advances.sciencemag.org/cgi/content/full/7/12/eaba7281/DC1

Supplementary Materials for

Deep learning velocity signals allow quantifying turbulence intensity

Alessandro Corbetta, Vlado Menkovski, Roberto Benzi, Federico Toschi*

*Corresponding author. Email: f.toschi@tue.nl

Published 17 March 2021, *Sci. Adv.* **7**, eaba7281 (2021)
DOI: 10.1126/sciadv.aba7281

This PDF file includes:

Sections S1 to S4
Figs. S1 to S6
Table S1

1 Viscosity estimates via structure functions

In this section we outline the derivation of the physical argument that we employ in order to estimate the viscosity. In particular, we derive the relation between the second order structure function, $S_2(\tau)$, in the small τ limit, and the viscosity ν. We apply the standard Eulerian-Lagrangian bridge relation to write the Lagrangian structure functions in the multi-fractal formalism. The velocity signals from the shell model of turbulence can indeed be regarded as a Lagrangian signal, due to the lack of sweeping. In this work we used data from shell model as well as data from Lagrangian passive tracers in fully resolved 3d DNS both signals should therefore be compared with the Lagrangian statistics.

In the inertial range, velocity differences, $\delta v(l) = v(r+l) - v(r)$, exhibit a singular, i.e. power law, behavior as a function of the observation scale l, i.e.

$$\delta v(l) \sim l^h, \tag{1}$$

for some $0 < h < 1$. Let τ_l the typical time-scale at scale l (i.e. $\tau_l \sim l/\delta v(l)$), then it holds

$$\tau_l \sim \frac{l}{\delta v(l)} = l^{1-h}. \tag{2}$$

The dissipative scale of the system (η, τ_η) satisfies

$$\frac{\delta v(\eta)\eta}{\nu} \sim 1, \tag{3}$$

or, in Lagrangian perspective,

$$\frac{\delta v(\tau_\eta)\tau^{\frac{1}{1-h}}}{\nu} \sim 1, \tag{4}$$

from which we have

$$\tau_\eta \sim \nu^{\frac{1-h}{1+h}} \qquad \text{and} \qquad \frac{\delta v(\eta)}{\tau_\eta} \sim \nu^{\frac{2h-1}{1+h}}. \tag{5}$$

Let $D(h)$ be the fractal co-dimension in which an h-singular behavior is observed, i.e. singularities with Hölder exponent h occur with probability

$$p(h) \sim l^{3-D(h)} = \nu^{\frac{3-D(h)}{1+h}}. \tag{6}$$

This enables to express explicitly structure functions as

$$\frac{S_2(\tau_\eta)}{\tau_\eta^2} = \frac{\langle \delta v(\eta)^2 \rangle}{\tau_\eta^2} = \int dh\, \nu^{\frac{3-D(h)}{1+h}} \nu^{2\frac{2h-1}{1+h}}, \tag{7}$$

which in the fully developed turbulence limit satisfies the power law

$$\frac{\langle \delta v(\eta)^2 \rangle}{\tau_\eta^2} = \nu^{-\alpha}, \tag{8}$$

where α is determined by a Legendre transform as

$$\alpha = \sup_h \frac{D(h) - 4h - 1}{1+h}. \tag{9}$$

The Kolmogorov K41 approach considers $D(h) = D = 3$, which yields $h = \frac{1}{3}$ and $\alpha_{K41} = \frac{1}{2}$. Conversely, employing the She-Leveque multifractal model for $D(h)$, yields $\alpha \approx 0.57$.

Inverting the relation in Eq. (8), in combination with the value of α, yields an estimator of the viscosity based on the second order structure function.

2 Width of the inertial range

In presence of limited statistics as in the case of relatively short signals, the estimation of the width of the inertial range or, similarly, the estimation of the viscosity, is enslaved to large scale energy fluctuations. On a time scale comparable to the large scale fluctuations, local increments or decrements of the system energy yield almost instantaneous widenings or shortenings of the inertial range. This effect can be naturally interpreted in terms of viscosity, where local energy increments play the same effect of a lower viscosity on the width of inertial range (see Figure S.1, where show this aspect for Eulerian structure functions).

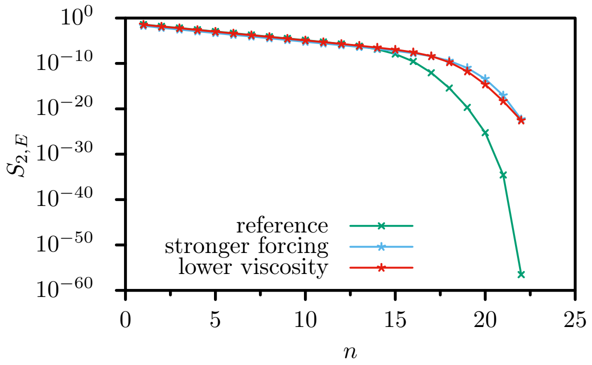

Figure S.1: **Impact of the variation of forcing (operating at the large scale, L) or viscosity (regulating the small scale, η) on Eulerian structure functions.** We compare a reference case with, respectively, a dynamics characterized by increased forcing (structure function translated and superimposed, *a posteriori*, to the reference), and a dynamics characterized by decreased viscosity. Both these two cases yield a higher Reynolds number wider extension of the inertial range.

In Figure S.2(a), we report Lagrangian structure functions for a set of training signals with fixed viscosity values. The limited statistics yield high fluctuations among the structure function, due to a combination of large-scale energy fluctuations and small scale intermittency. In Figure S.2(b), we amend large-scale fluctuations by normalizing by the signal energy, i.e. we report $S_2(\tau)/S_2(\infty)$.

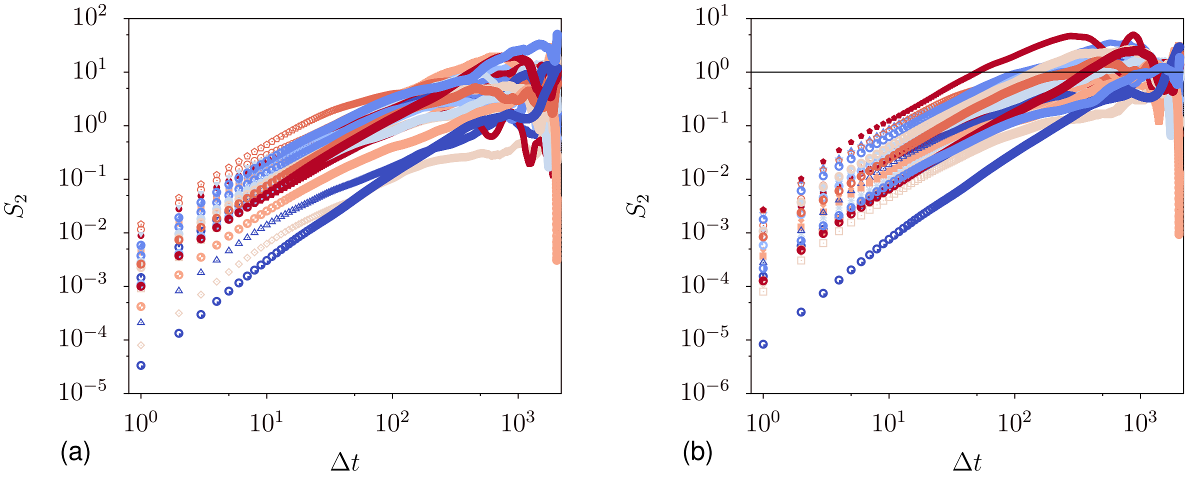

Figure S.2: **Collection of second order Lagrangian structure functions, S_2, without (a) and with (b) normalization with respect to the integral scale energy, i.e. the asymptotic value $S_2(\infty) = 2v_{rms}^2 \approx S_2(T)$.** Each plot reports a collection of 25 structure functions extracted from the training set and with associated viscosity $\nu = 0.0005$. The x-axis is in units of sampling time, Δt, as presented to the DNN.

3 Data generation, training, testing and neural network parameters

We include in Table 1 the parameters considered in the shell model simulations by which the training, validation and test datasets have been created. In Figure S.3 we complement Figure 1 by including, for the same three viscosity levels, further features of the considered signals. These are: (a) second order Eulerian structure functions, $S_E^2(n)$ (where $S_{p,E}(n) = S_{p,E}(k_n) = \langle |u_n|^p \rangle$) showing that changing the viscosity only affects the extension of the inertial range; (b) relevant time scales (computed by inertial scaling) associated with the dynamics of the different shells; (c) signals energy as a function of time. In Figure S.4, we report the diagram of the neural network. Relevant structural parameters (e.g. size of the convolutional filters) are reported in the figure caption.

Parameter	Value	
N	28	Number of shells
k_0	0.05	Wave number integral scale
λ	2	Inter-shell distance
$\sigma(f_0)$	2	Noise intensity forcing on shell 0
$\sigma(f_1)$	$2/\sqrt{2}$	" on shell 1
dt	$5 \cdot 10^{-5}$	integration step
Δt	$1000\,dt$	sampling time DNN
T	$2048\,\Delta t$	length window DNN

Parameter	Training	Testing
min(ν)	$2.5 \cdot 10^{-5}$	$6.0 \cdot 10^{-5}$
max(ν)	$9.75 \cdot 10^{-4}$	$9.6 \cdot 10^{-4}$
increment ν	$2.5 \cdot 10^{-5}$	$6.0 \cdot 10^{-5}$
levels	39	16
set size	192.000	6.600
training:validation ratio	75%:25%	N/A

Table 1: (Left) Relevant parameters for the shell model its numerical integration; time length and sampling of the signals as provided to the deep neural network (DNN). (Right) Viscosity values considered for training validation and test; size of the related datasets.

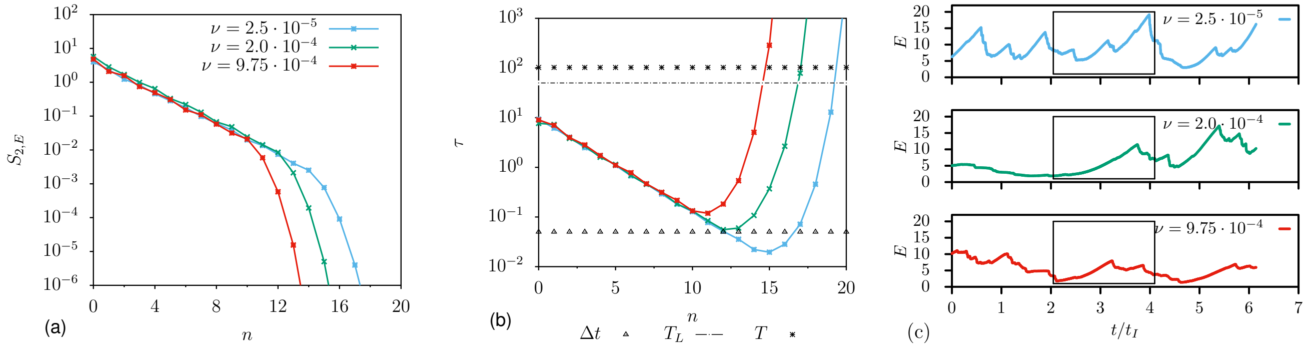

Figure S.3: **Comparison of shell model output for the three viscosities reported in Figure 1.** (a) Eulerian structure functions $S_{2,E}(n) = \langle |u_n|^2 \rangle$. Reducing the viscosity leads to an extension of the inertial range, while the energy content of the larger scales remains unchanged. (b) A scale-by-scale estimate of the correlation times for the shell models (via the inertial scaling $\tau_n \sim (k_n u_{n,rms})^{-1}$) for three different viscosity values. The observation window T, the calculated decorrelation time of the integral scale T_L, and of the DNN sampling time Δt are reported. (c) Energy time-histories for the signals reported in Figure 1.

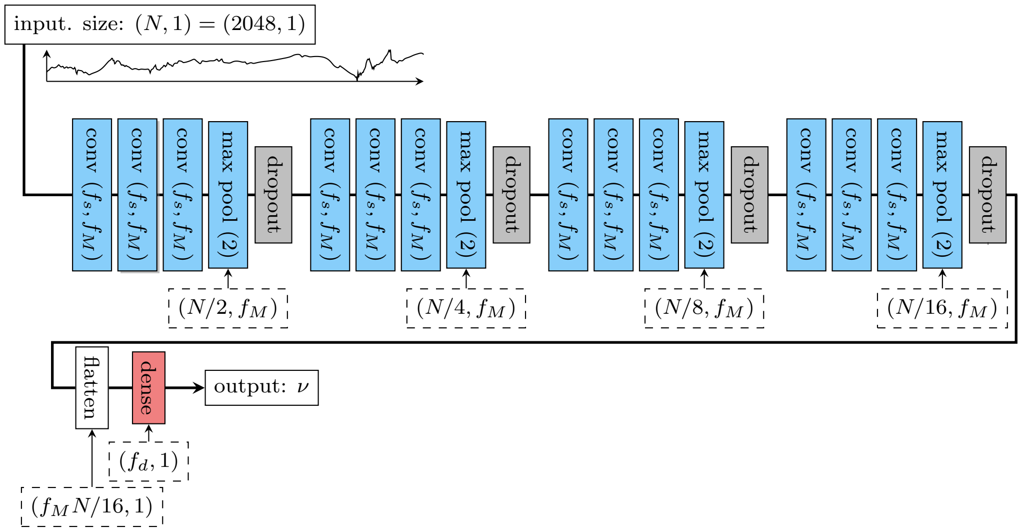

Figure S.4: **Feed-forward convolutional neural network considered.** The network is constituted of four blocks each encompassing three convolutional layers ("conv", filter size $f_s = 3$, filter number $f_M = 128$, activation function: Re-Lu) one max pool layer that down scales the signal by a factor two and, in training, a dropout layer with dropout probability 20%. The dimensions of the feature map as obtained at the end of each block is reported in the dashed rectangles. The last feature map (dimension $(128, 128)$), is densely connected to a representation layer which has $f_d = 128$ dimensions and Re-Lu activation. The final output, i.e. the predicted viscosity ν, is built from a linear combination of the dense representation values.

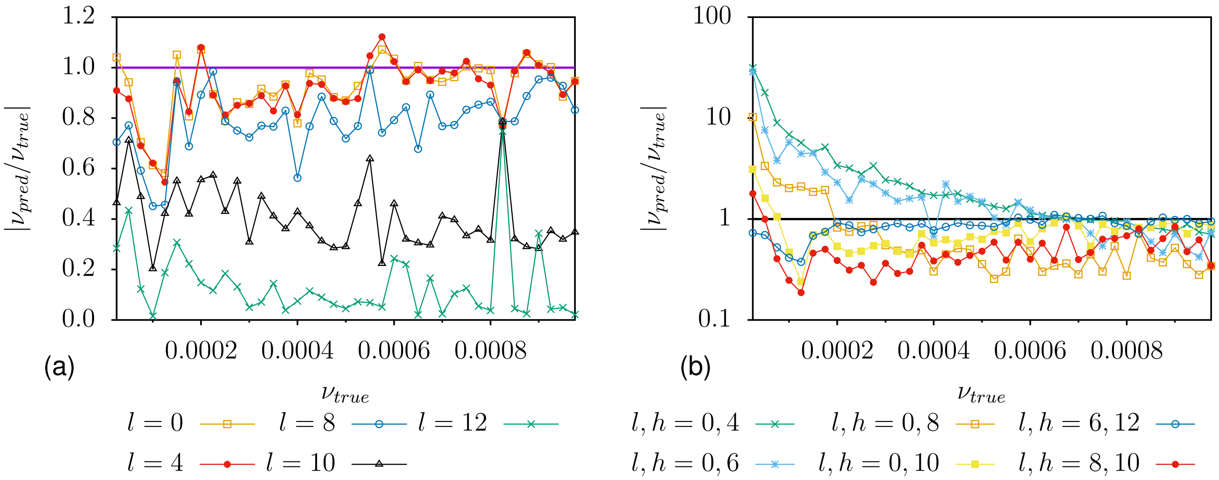

Figure S.5: **Viscosity predictions with ablated input signals from (a) an highpass filter:** $v(t) = Re \sum_{n=l}^{26} u_n(t)$**, (b) a bandpass filter:** $v(t) = Re \sum_{n=l}^{h} u_n(t)$**.** In both cases, one single sample signal is considered for each viscosity value. Predictions are reported normalized with respect to the true value.

4 Features observed by the DNN

During training, the DNN develops feature detectors. As discussed in the main text, we expect these detectors to select features that, at the same time, strongly correlate with the turbulence intensity and that are insensitive to large scale oscillations. As generally expected in deep learning, detectors are likely specific to the parameter range and statistical properties of the signals contained in the training set.

In this section, to understand the characteristics of the signal that our model relies on, we develop an ablation study by systematically altering the content of randomly selected testing signals. The modifications considered involve the suppression of frequency components, or the random shuffling of the time structure. This enables us to identify features mostly ignored by the DNN and, conversely, restrict the set of characteristics of the signals relevant for the DNN.

In Figure S.5 we consider testing signals that have been altered through a high-pass (a) or a band-pass filter (b). In the case of Lagrangian signals, filtering operations are easily performed by restricting the summation in Eq. (2) to a subset of the shell signals. We select one testing signal per viscosity level, we ablate its spectral structure and we plot the DNN prediction. We notice that the neural network is almost insensitive to the large scale dynamics, as the estimates after the high-pass filter remain unaltered if the large-scale shells are removed. We notice, in particular, that any selection of a band of shells that includes the last part of the inertial range yield almost error-free predictions.

Similarly, we can alter the time structure of the signals by partitioning them in disjoint contiguous blocks of length T_B, and then by randomly mixing these blocks. In Figure S.6 we report the predictions for different block extensions. As the block extension remains in the same order of the integral scale, the prediction remain mostly unaltered, to then degrade as the block size become comparable to the dissipative time-scale. This shows how the training develop feature extractors targeting fine scales and correlations existing around the dissipative end of the inertial range.

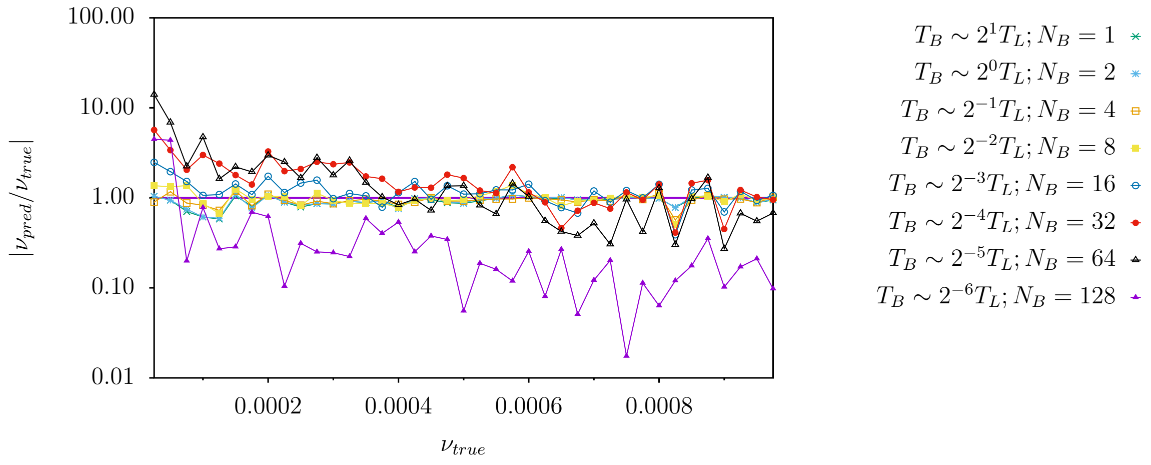

Figure S.6: **Viscosity predictions for block-based time-altered signals.** Alteration is performed by splitting an initial signal in N_B blocks (with time length T_B reported in terms of the integral time scale) and then by performing a random permutation of the blocks. One single sample signal is considered for each viscosity value. Predictions are reported normalized with the true value.
